# Evaluation of Polycaprolactone Electrospun Nanofiber-Composites for Artificial Skin Based on Dermal Fibroblast Culture

**DOI:** 10.3390/bioengineering9010019

**Published:** 2022-01-06

**Authors:** Morshed Khandaker, Hembafan Nomhwange, Helga Progri, Sadegh Nikfarjam, Melville B. Vaughan

**Affiliations:** 1 Department of Engineering & Physics, University of Central Oklahoma, Edmond, OK 73034, USA; hnomwhange@uco.edu (H.N.); hprogri@uco.edu (H.P.); 2 Center for Interdisciplinary Education and Research, University of Central Oklahoma, Edmond, OK 73034, USA; MVaughan4@uco.edu; 3 Department of Biology, University of Central Oklahoma, Edmond, OK 73034, USA; snikfarjam@uco.edu

**Keywords:** hydrogel, polycaprolactone, dermal equivalent model, electrospun, nanofiber

## Abstract

The study’s aim was to develop a dermal equivalent scaffold that can mimic the architecture and biological performance of the human dermis. Poly ε-caprolactone (PCL) electrospun nanofiber material (ENF) was assembled with polyethylene glycol diacrylate (PEGDA), sodium alginate (SA) and type I collagen (CG1) to develop three groups of dermal equivalent scaffolds. These scaffolds were named PEGDA-PCL, SA-PCL and CG1-PCL. Scanning electron microscopy (SEM) images of cell-free scaffolds’ top and cross-sectional surface were collected and analyzed to examine internal morphology, specifically the adhesiveness of PCL fibers with the different scaffolds. Human dermal fibroblasts were cultured on each of the scaffolds. Cell viability studies including cell adhesion, cell differentiation and stress fiber production were conducted on each scaffold. Furthermore, the architectural integrity of each scaffold was verified by degradation analysis for 2 weeks by soaking each scaffold in phosphate-buffered saline (PBS) solution. Finally, we conducted rheological characteristics of each scaffold. Based on our results from the above analysis, the study concluded that CG1-PCL is best suitable for the dermal equivalent model and has potential to be used as a graft for skin repair.

## 1. Introduction

### 1.1. Dermal Equivalent Scaffold

The skin is the largest organ in the body. It is one of the most important organs of the body due to its numerous functions. It regulates homeostasis, performs sensory functions, and protects the body from physical damages due to assault. Damaged skin results in loss of function, leading to various complications such as loss of fluid or infections [1]. Non-healing wounds present a significant and increasing problem in the health care system [2]. While much is known about biological processes involved in wound repair and maintenance of skin structure and integrity, we lack understanding of how novel bioengineered materials can facilitate these processes. In vitro cell-based studies can help provide fast and simple material evaluation prior to animal studies. In this research, three different dermal scaffolds were created and their characteristics examined to evaluate their potential to be used for wound-healing skin equivalent scaffolds. 

### 1.2. PEGDA, Alginate and Collagen as Dermal Equivalent Scaffold Materials

Hydrogel scaffolds can be tuned to resemble dermal tissues’ mechanical and biological properties [3]. Polyethylene glycol diacrylate (PEGDA) is a photosensitive hydrogel. It is possible to make any shape of skin graft using PEGDA. In our previous research, PEGDA hydrogel scaffolds were developed for the reconstruction of intervertebral disc and cartilage repair [4]. Although the in vivo performance of PEGDA hydrogel scaffolds has not yet been reported, our pilot study using a rat-tail model showed the suitability of using the PEGDA for soft tissue repair [5]. Collagen (especially type I) is a fibrous protein formed from a triple-helix of polypeptides that provides strength to connective tissues [6]. Alginate has numerous applications in biomedical science and engineering due to its excellent biocompatibility and ease of gelation [7]. Alginate hydrogels have been used in soft tissue engineering applications, including wound healing and drug delivery [8,9,10]. Alginate gels retain structural similarity to the extracellular matrices in soft tissues and can be manipulated and grafted to reconstruct skin [11]. Sodium alginate (SA) hydrogel, one of the class of alginates, has been used as an engineering scaffold for soft tissue grafting [12]. SA hydrogels can effectively be made from sodium alginate and a calcium chloride solution [11] that, when combined, becomes a soft clear gel over time. The adequate porosity of the SA hydrogel allows for three-dimensional cell growth [9]. For example, cell-rich hydrogels can be introduced into the body to replace cartilage and help the body heal [13]. Collagen I (CG11) is an integral part of the extracellular matrix (ECM). It is synthesized and secreted by fibroblasts to stimulate faster wound healing [14]. It is the most abundant natural protein produced not only in the skin tissue but overall in the human body [15]. CG1 is predominantly a part of the ECM of skin, tendon, ligament, and bone. Therefore, PEGDA, SA and CG1 are suitable materials for dermal equivalent scaffolds. 

### 1.3. Polycaprolactone (PCL) Electrospun Nanofiber Mesh (NFM) for Tissue Engineering

**Figure 1:**
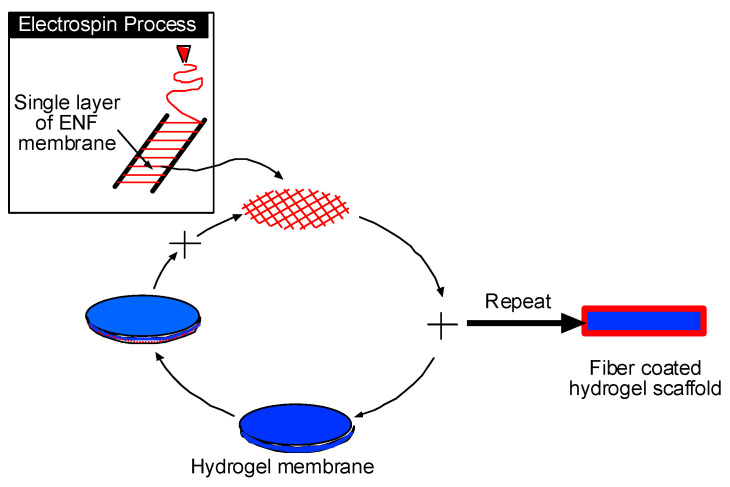
Schematic representation to produce a nanofiber-coated hydrogel scaffold.

Polycaprolactone (PCL) is a non-toxic, biodegradable polymer with good biocompatibility. PCL is one of the ideal materials for preparing nanofibers using an electrospinning process that provides it with high biocompatibility [16]. Electrospinning is a process by which fibers (micro to nanometer diameter) can be obtained from an electrostatically driven jet of polymer solution through a charged needle [17]. The high surface area-to-volume ratio of the fiber matrix provides an advantage in industrial applications such as barrier fabrics, wipes, and other medical/pharmaceutical uses [18,19,20,21]. In our previous research, we functionalized PCL with CG1 to increase its biological functionality [22]. We have developed a process [23] to create a membrane where multiple layers of aligned polycaprolactone (PCL) electrospun nanofibers (ENF) are harvested and applied to any substrate [23]. Two parallel wires were used to collect a single layer of aligned PCL fibers by electrospinning a PCL solution (Figure 1). The fibers were harvested at different angles and stacked in layers on a substrate to produce a PCL nanofiber membrane (NFM). According to our developed method, hydrogel membranes can be coated on all sides using PCL NFM to produce a PCL-coated hydrogel scaffold. The unique nature of the nanofiber-hydrogel scaffold is that any custom size and shape of tissue engineering graft (such as ear, nose, lips, or skin) can be produced from this method. Designing and developing pro-angiogenic electrospun PCL nanofibers and evaluating their usefulness in various tissue engineering and regenerative medicine settings is an active field of research [24]. Bazzolo et al. [25] conducted breast cancer cell cultures on electrospun PCL as a potential tool for preclinical studies on anticancer treatments. Various electrospun nanofibers made of natural and synthetic polymers are often used to incorporate biomolecules and peptides to improve the physico-chemical/mechanical and biological properties of PCL fibers. Abebayehu, D. et al. [26] reported improvement of angiogenic responses by adding vascular endothelial growth factor (VEGF), basic fibroblast growth factor (bFGF) with PCL. The study by Dettin M et al. [27] investigates the influence of increasing concentrations self-assembling peptides (SAP) with PCL on the physico-chemical, mechanical and biological properties of PCL fibers. The study found that, as compared with bare PCL, SAP enrichment increased the number of metabolic active h-osteoblast cells, fostered the expression of specific osteoblast-related mRNA transcripts, and guided calcium deposition, revealing the potential application of PCL-SAP scaffolds for the maintenance of osteoblast phenotype [25].

### 1.4. The Current Problem of Hydrogel Alone as a Dermal Equivalent and Its Solution

The fiber material, fiber diameter, fiber alignment in the membrane and thickness of the membrane control the biological functions of the membrane [28]. The fiber diameter in the ENF membrane is usually less than 500 nanometers. For a membrane with multiple layers of fibers, the thickness of the membrane can be larger than micrometers. When membrane layer thickness is in the micrometer range, the biological functions of the membrane decrease significantly due to the loss of porosity; porosity loss is negatively correlated to membrane thickness. Conversely, hydrogel scaffolds have limited mechanical properties such as stiffness and elasticity [29]. The biological efficiency of a hydrogel depends upon its thickness. It is possible to improve the properties of thick hydrogels by creating membranes composed of multiple and alternating layers of nanofiber and hydrogel. A nanofiber matrix-incorporated hydrogel is a composite polymer in a network form. The composite scaffold that is constructed by layers of hydrogel and PCL can overcome the individual limitations of PCL, ENF, and hydrogel scaffold. The combination of favorable properties of each hydrogel (PEGDA, SA and CG1), with PCL, results in a new hybrid system (PEGDA-PCL, SA-PCL and CG1-PCL) with properties that can significantly improve or substantially differ from those of the individual polymers or from each other. Therefore, we compared the physical, bio-adhesive, and degradative properties of PEGDA-PCL, SA-PCL and CG1-PCL in this study to evaluate their relative qualification for a dermal equivalent model.

## 2. Materials and Methods

### 2.1. Materials

To prepare PEGDA hydrogels, we used polyethylene glycol diacrylate (PEGDA), (M_n_ 700 mol, Sigma), Dulbecco’s phosphate buffered saline (DPBS), alpha-alpha-dimethoxy-alpha-phenylacetophenone (MW = 256.35 g/mol), and 1-vinyl-2-pyrrolidone (MW = 111.14 g/mol). These chemicals were purchased from Sigma-Aldrich, St. Louis, MO, USA. To prepare alginate hydrogels, we used sodium alginate (Protanal^®^ LFR 5/60, FMC BioPolymer (Philadelphia, PA, USA)), Dulbecco’s Modified Eagle Medium (Thermo Fisher) and calcium chloride. To prepare CG1 hydrogels, we used Type I bovine collagen (Gibco, Waltham, MA, USA), phosphate buffered saline solution (PBS). Calcium chloride and NaOH were collected from a chemistry laboratory at the University of Central Oklahoma. To produce PCL fibers, we used PCL beads (pellet size ~3 mm, average M_n_ 80,000) and acetone (laboratory reagent ≥ 99.5%). Both PCL pellets and acetone were purchased from Sigma Aldrich, St. Louis, MO, USA).

### 2.2. Sample Preparation

#### 2.2.1. Electrospun Nanofiber Mat

**Figure 2:**
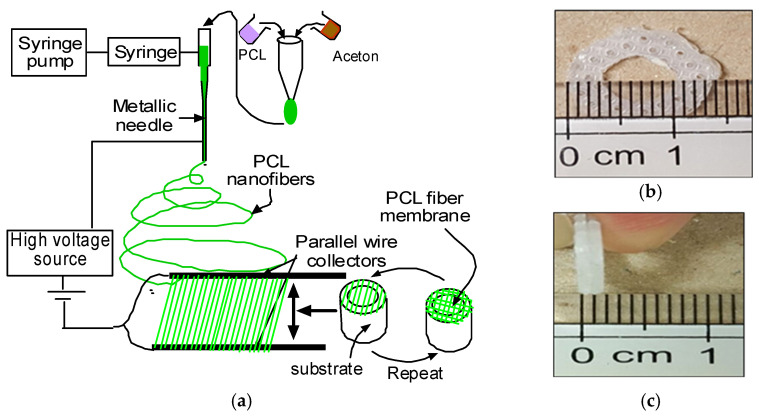
(**a**) Schematic representation of production of parallel nanofibers and collection of PCL fiber membrane on a round shape collector. Inset picture shows the PCL fiber on the collector. A plastic ring was used as an anchor for mechanical tension development. (**b**) Front view and (**c**) side view.

During this study, three groups of dermal equivalents were prepared. They were PEGDA PCL, SA-PCL and CG1-PCL. To make PCL fiber mats for all dermal equivalents, PCL solution was made by ultrasonic mixing of 7.69 wt% of PCL pellets of size approximately 3 mm and average molecular number 80,000 using the protocol published in our earlier work [22]. In short, the PCL and acetone solution was sonicated for 45 min and then poured into a plastic syringe in an infusion pump (Harvard apparatus). The PCL fibers were then ejected from the syringe through a 1-inch discharge metallic needle (aluminum 23G blunt needle, Model # BX 25). The needle was charged with a 9-kilovolt power supply (Gamma High Voltage, model # ES 30). Aligned fibers were collected using a wooden block with two parallel electrodes attached to it. Twenty-four layers of fibers were collected manually on an acrylic mold through repetition of forward and backward motions; each layer of fiber was collected at a right angle to the next layer (Figure 2a).

#### 2.2.2. PEGDA-PCL Scaffold

**Figure 3:**
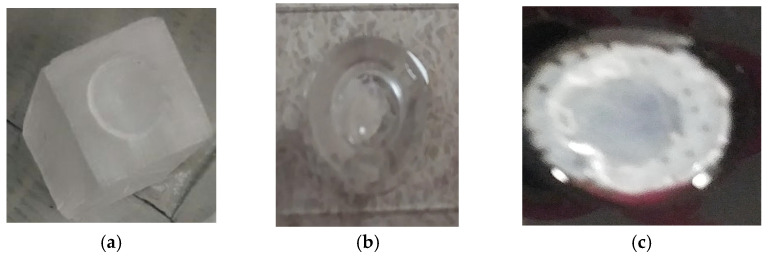
(**a**) PCL NFM coated PEGDA scaffold on an acrylic mold; (**b**) Cured SA gel on microscopy slide; (**c**) A PCL coated CG1 sample on a plastic ring.

PEGDA-PCL dermal equivalents (Figure 3a) were made as follows. The activated photo initiator mixture was made by mixing 0.3 g of photo initiator (alpha-alpha-dimethoxy-alpha-phenylacetophenone) per each milliliter of vinyl-2-pyrolidinone. Two mL of PEGDA were mixed with eight mL of 1× PBS to make a 20% PEGDA mixture. 4 microliters of activated photo initiator mixture was combined with two mL of 20% PEGDA which was cured for 3 min in room temperature to form the semi-solid PEGDA. Each PEGDA gel (approximately 0.5 mm thickness, 10 mm diameter) was sandwiched between 24 layers of PCL fiber membranes. Cells were then plated and cultured as described below.

#### 2.2.3. SA-PCL Scaffold

To make SA-PCL dermal equivalents (Figure 3b), sodium alginate powder was weighed to 220 g and added to 10 mL of deionized water using a 50 mL beaker; this was stirred by hand for 3 min; a sonicating machine was used to thoroughly mix for 5 min with 2-min hand mixing intervals; this was mixed until powder was fully dissolved in water. Calcium chloride (110 g) was added to 5 mL of water in a 50 mL beaker; this was stirred by hand for 3 min; the sonicating machine was used to thoroughly mix for 5 min with 2 min hand mixing intervals, this was mixed until the powder was totally dissolved in water. Both solutions were then mixed together using the sonicating machine. Sodium alginate gel was produced by pipetting 80 µL of solution into silicon molds; these silicon molds were incubated at −20 degrees for 2 h and then thawed for 1 h. Each SA gel (approximately 0.5 mm thickness, 10 mm diameter) was sandwiched between 24 layers of PCL fiber membranes. Cells were then plated and cultured as described below.

#### 2.2.4. CG1-PCL Scaffold

To make CG1-PCL dermal equivalents, bovine collagen (GIBCO, Thermo Fisher) was mixed with ultrapure water and a premix concentrate of media salts, sodium bicarbonate, and fetal bovine serum as previously published [30,31]. First, PCL fibers were ejected from a glass syringe through a discharge needle. Aligned fibers were collected using two parallel electrodes similar to the above procedure. A total of 24 layers of fibers were collected manually on an acrylic mold through repetitive back and forth motions (Figure 3a). Each layer of fiber was collected at a right angle to the next layer. PCL fibers were placed on a plastic tension ring (Figure 3b). Collagen mixture was prepared, and pH equilibrated with 1N NaOH as needed, then human fibroblasts resuspended in growth media were mixed with the collagen, yielding a final concentration of 1 mg/mL collagen and 200,000 fibroblasts/mL. A plastic ring used to maintain tissue tension was produced as follows: a stiff plastic canvas sheet, 14-mesh was purchased from an art supply or hobby store (for example, Darice stiff mesh; Jo-Ann Fabric). The mesh was punched with a #7 cork borer (Fisher Scientific) to produce the outer edge of each ring, while the inner opening was punched with a #4 borer. Sharp edges were smoothed with a file or sandpaper. Each ring was rinsed with deionized water and sterilized with 70% ethanol and stored in sterile HBSS until use. The plastic tension ring was inserted into the bottom of a well of a 24-well plate, and PCL fibers added atop the ring, and warmed on a slide warmer to 37 °C. One milliliter of collagen–cell mixture was pipetted into the well atop the PCL fibers and left on the slide warmer until the collagen began to polymerize (about 10–15 min). The CG1-PCL dermal equivalents (Figure 3c) were placed in a CO_2_ cell culture incubator for 72 h in the presence of 1 mL growth media per equivalent.

### 2.3. Experiments and Analysis

#### 2.3.1. SEM Examination

This study used a Hitachi™ (Chiyoda, Tokyo, Japan) 3000 scanning electron microscope (SEM) to observe the quality of fiber embedding and internal morphology in each group of hydrogel. SEM images were captured on the top fiber layer surface to observe fiber integration with the hydrogel, whereas cross-section images of paraffin-embedded scaffold samples were examined to observe internal void or porosity. For SEM histology sections, each scaffold was made without cells and inserted into optimal cutting temperature fluid (OCT) using tweezers. The procedure was performed carefully to prevent any air bubbles from forming in the fluid. Tissues embedded in OCT were chilled to −20 °C to harden samples for frozen sectioning using a cryostat (Leica CM1800). Thin sections of tissues were collected onto room temperature microscope slides and stored at −20 °C. SEM images were captured from all sections. 

#### 2.3.2. Cell Culture and Assays

This study used human dermal fibroblasts (LifeLine Technology, Frederick, MD, USA) HDF 01035). Cells were cultured on tissue culture dishes in standard culture conditions (37 °C in a 5% CO_2_ incubator). The cell medium consisted of DMEM/high glucose, 5% FBS and 1% penicillin/streptomycin/amphotericin (Sigma-Aldrich, St. Louis, MO, USA) was used for all studies. Cells were extracted from the dish using 1× trypsin/EDTA solution (Sigma Chemical) for 5 min at room temperature. Cell extraction was followed by serum inactivation. Cells were embedded within the collagen in CG1-PCL tissues; CG1-PCL dermal equivalents were cultured submerged for 7 days to allow cells to remodel the collagen to provide sufficient tension and stiffness for proliferation and differentiation [32]. In PEGDA-PCL and SA-PCL, fibroblasts were plated on the upper surface using an 8 mm borosilicate glass cloning cylinder; for each sample, 60,000 fibroblasts were resuspended in 75 microliters of media and added to the cloning cylinder sitting atop the tissue. The tissues and cylinders were returned to the incubator for 2–4 h to allow fibroblasts to settle and attach to the upper surface of the tissues, after which time the cylinders were removed and tissues were submerged in growth media for 7 days. Cell adhesion, proliferation, differentiation and focal adhesion tests on each of the dermal equivalents was conducted according to the method established in our previous research [32,33]. Click-iT EdU Alexa Fluor 488 Imaging Kit from Thermo Fisher Scientific (Waltham, MA, USA) was used to evaluate proliferation for each sample [32,33]. The assay required addition of a tagged nucleotide to the culture media during the last 1–24 h of culture; proliferating cells incorporated EdU into the replicated DNA during this time [32]. For histology, after a total of 7 days, dermal equivalents were fixed with paraformaldehyde, permeabilized with MeOH and stained to identify proliferation and differentiation in a single combined stain, similar to [32]. Each group of samples was stained for 1 h with mouse anti-human vimentin (Labvision; Thermo Fisher) for one hour, followed by a secondary vimentin with goat anti-mouse rhodamine (Thermo Fisher Molecular Probes), followed using click chemistry (as per manufacturer’s instructions) and DAPI counterstain. To identify focal adhesion structures, each group of samples was cultured in the same conditions as for the proliferation and differentiation stains; however, upon harvest the samples were fixed using 3% paraformaldehyde and 0.2% Triton-X-100 prior to staining [34]. Samples were stained using mouse anti-human vinculin (Clone VIN-1; Millipore Sigma) and goat anti-mouse Alexa 488, followed by rhodamine phalloidin (Thermo Fisher) staining to label actin stress fibers. Stained whole-mount samples were inverted onto large coverslips for visualization using 100× oil immersion on an Olympus IX-71 microscope. Images were captured using an Olympus DP72 camera equipped with CelSens software. Subsequent quantification was performed using Image J software using a published protocol [35] with modifications.

#### 2.3.3. Degradation Analysis

Each scaffold weight was measured immediately upon fabrication using a precision balance, then soaked in PBS for 24 h, 7 days and 14 days. After each period of soaking, the weight of the scaffold was measured. The value of the rate of degradation was measured using the formula: (W_t_ − W_0_) × 100%/W_0_. In this equation, W_0_ is the weight of the scaffold after 24 h of soaking in PBS and W_t_ is the weight of the scaffold after each time period of soaking in PBS.

#### 2.3.4. Rheological Evaluation

Dynamic compression tests were carried out following the procedures described in our earlier research [36]. Frequency sweep tests (0.1–10 Hz) at 25 °C at constant strain rate were performed on each sample group using the Netzsch Instruments KINEXUS Rotational Rheometer. Elastic, viscous and phase shift angle at 1 Hz was compared among the sample group.

#### 2.3.5. Statistical Analysis

This study used Microsoft Excel statistical tools to test statistical differences in mean values of cell adhesion, proliferation, differentiation, and degradation among the sample groups. The study conducted independent sample *t* tests, assuming unequal variances to find an appropriate statistical significance level.

## 3. Results

### 3.1. Scanning Electron Microscopy

**Figure 4:**
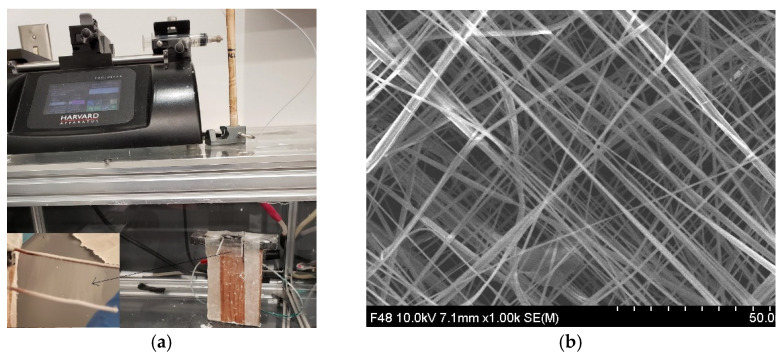
(**a**) Fabrication of random 24 layers of PCL fibers for coating the gels using the electrospinning process. Inset picture shows the collected single layer of aligned fibers between two-wires in the electrospun machine which was used to fabricated 24 layers of PCL fiber mesh. (**b**) SEM image of 24 layers of PCL fiber mesh.

Figure 4a shows the 24 layers of fiber mesh that were collected from a single layer of fiber mesh. The direction of the 24 layers of PCL deposition on the collector was mainly random as seen from the SEM images of the membrane (Figure 4b). The fiber diameter in the mesh was in the range of 300 to 500 nm. The alignment of single layer-deposited fibers between two wires was unidirectional (Figure 4b). This study used 24 unidirectional layers of PCL fibers to coat PEGDA, SA and CG1 hydrogels. 

**Figure 5:**
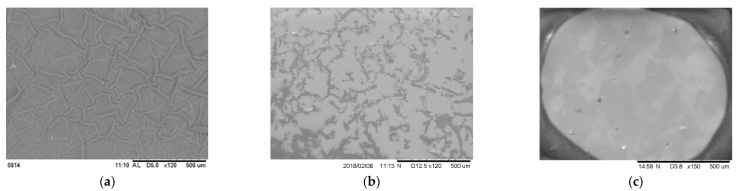
Surface topographical images of the (**a**) PEGDA-PCL, (**b**) SA-PCL, and (**c**) CG1-PCL specimen collected using a scanning electron microscope. The scale bar for all images is 500 µm.

A clearly visible difference in surface (Figure 5) was observed from SEM images among different groups of dermal equivalent samples. This result suggested a significant change of the surface texture due to the gel architecture and PCL NFM treatment. The overlapping 24 fiber layers were fully embedded in each gel. 

**Figure 6:**
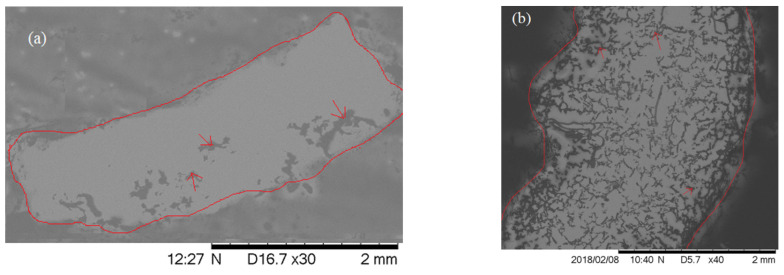
Cross-sectional images of the (**a**) PEGDA-PCL and (**b**) SA-PCL hydrogels. The red line shows the boundary between embedding media and scaffold. The histology section thickness for both samples was 5 microns. Each image was captured 30× magnification. The arrows show the presence of voids.

It is clear from comparing the cross-sectional SEM images between PEGDA-PCL and SA-PCL that there is a visible difference in porosity among the samples (Figure 6). There is a large size of void created in PEGDA-PCL (Figure 6a), whereas the void size is small in SA-PCL samples (Figure 6b). PCL fibers were easily visible in PEGDA-PCL, while the fiber could not be distinguished in the SA-PCL samples. Cross-sectional image of the CG1-PCL sample was not possible due to the structure of CG1 hydrogel. Since CG1 solution contains acetic acid, the CG1 solution evaporates and loses integrations with PCL fiber quickly before freezing for microtome sectioning.

### 3.2. Cytocompatibility Properties 

**Figure 7:**
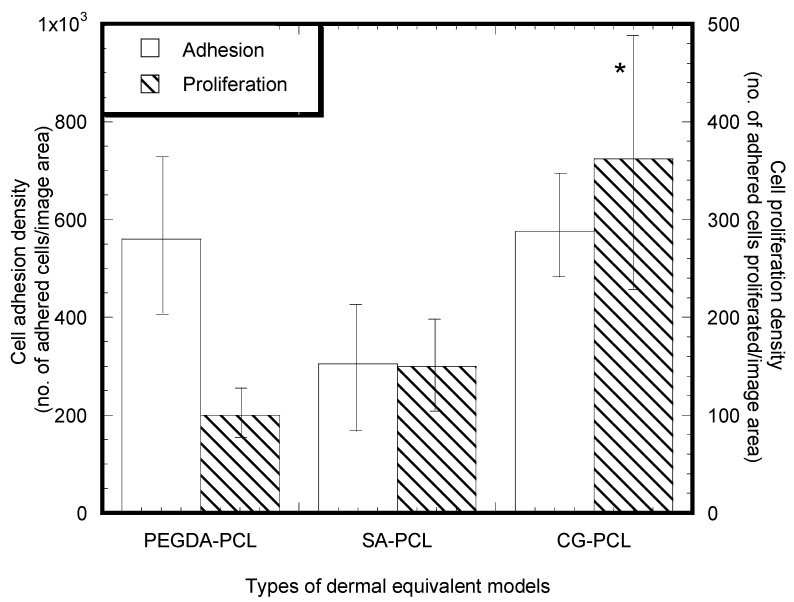
Mean cell adhesion density (±standard error) and mean cell proliferation density (±standard error) for the PEGDA-PCL, SA-PCL, and CG1-PCL groups. Data are presented with *n* = 8 for all samples. Note: * *p* < 0.05 (compared to PEGDA-PCL).

Immunostained whole-mount images of PEGDA-PCL, SA-PCL, and CG1-PCL specimens indicated cell presence, differentiation, and proliferation on each of the different kinds of hydrogel. The images indicated the success of cell attachment, proliferation and differentiation in PEGDA-PCL, SA-PCL, and CG1-PCL. Cells were better able to proliferate and differentiate in the CG1-PCL scaffold compared to PEGDA-PCL and SA-PCL (Figure 7). Although this study did not find any significant difference of cell nuclei adherence and proliferation density among different groups of samples (*p* > 0.05), the mean number of cells proliferated after adherence was higher for the CG1-PCL samples compared to the other samples. A large value of standard of error was found for each assay. There was no significant difference in the mean amount of adherence as measured by presence of cell nuclei (total blue stained area × 100%/area of image field) among sample groups (*p* > 0.05) (Figure 7).

**Figure 8:**
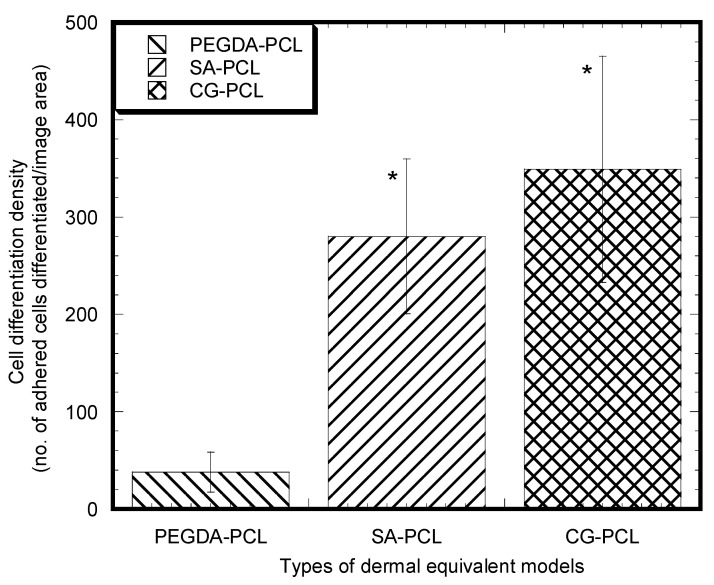
Mean cell differentiation value (±standard error) for the PEGDA-PCL, SA-PCL, and CG1-PCL groups. Data are presented with *n* = 8 for both samples. Note: * *p* < 0.1 (compared to PEGDA-PCL).

There was a significant difference in the mean amount of cell differentiation (total vimentin stained area × 100%/area of image field) between the PEGDA-PCL and SA-PCL sample groups (*p* value = 0.05) as well as the PEGDA-PCL and CG1-PCL sample groups (*p* value = 0.07) (Figure 8). There was no significant difference in the mean amount of cell differentiation in SA-PCL and CG1-PCL sample groups. The result suggested that the inclusion of PCL in SA and CG1 has a higher effect on cell differentiation compared to the inclusion of PCL in PEGDA.

**Figure 9:**
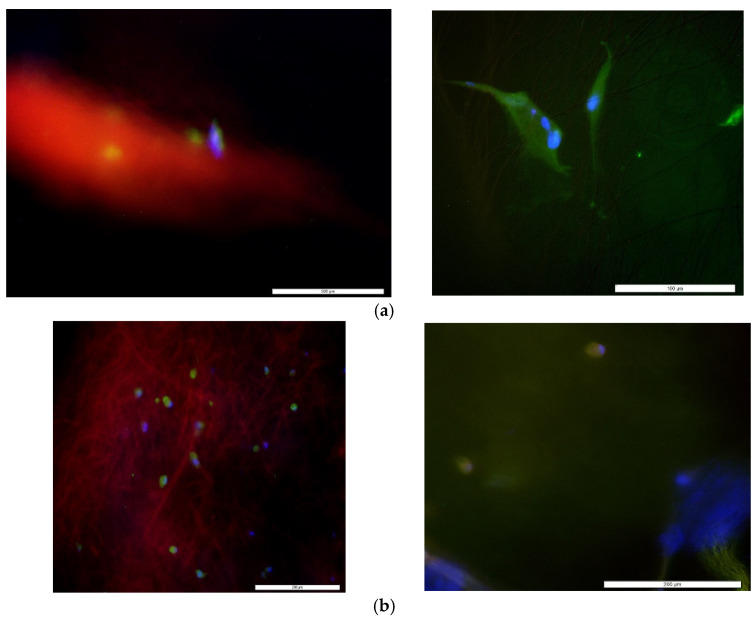

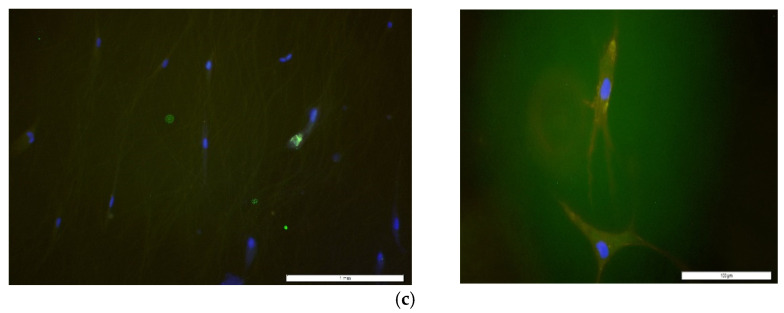
A representative fluorescent stained images (left) showing the existence of stress fibers and elongated cells (right) in (**a**) PEGDA-PCL, (**b**) SA-PCL and (**c**) CG1-PCL dermal equivalent models. All images were captured at 100× magnification. Scale bar in each image is 200 μm.

Stress fibers successfully occurred on the surface of all types of dermal equivalents as shown in Figure 9. All samples demonstrated well-spread cells based on the stress fiber and focal adhesion stains. The results confirm that cells in the dermal equivalent produced with hydrogel and PCL fibers have normal mechanotransduction characteristics (the mechanism by which cells convert attachment and stiffness into mechanical tension).

### 3.3. Degradation Analysis

**Figure 10:**
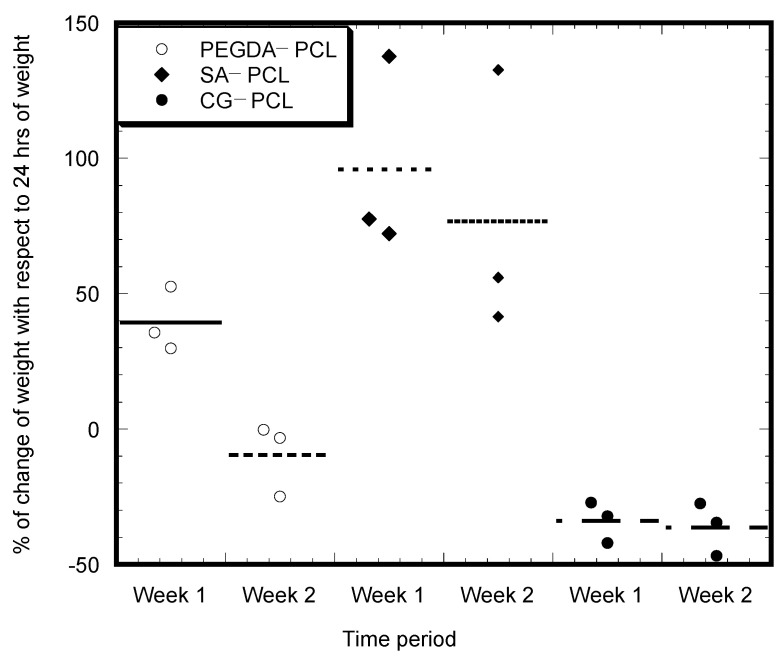
Percentage of weight change plots of the PEGDA-PCL, SA-PCL, and CG1-PCL specimens with respect to 7 and 14 days swelling in PBS. The values of the weights were normalized with respect to weights of the samples after swelling the samples for 24 h. Data are presented with *n* = 3 for all samples.

The CG1-PCL scaffold demonstrated a consistent degradation from the first week, whereas there was an increase of weight due to absorption of water for the SA-PCL scaffold during the two-week study period (Figure 10). The study observed degradation of PEGDA-PCL scaffold during the second week. Unlike PEGDA-PCL and SA-PCL, CG1-PCL did not absorb fluid during the period of degradation analyses. However, degradation data indicate a decrease of architectural integrity with time. All samples showed degradation from the 1st week to the 2nd week, with the highest values for PEGDA-PCL samples, then SA-PCL. There is a negligible amount of degradation observed for CG1-PCL samples during the 2-week soaking period.

### 3.4. Rheological Characterization 

**Figure 11:**
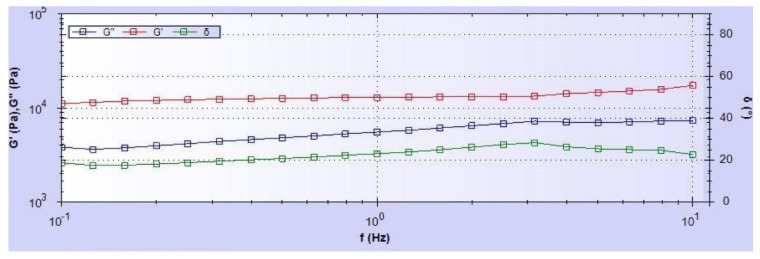
A representative image showing the elastic, viscous and phase shift angle variation during the constant strain rate frequency sweep test at 25 °C. In the image, G′, G″ and δ are the elastic, viscous and phase shift angle, respectively.

There is a significant difference among the rheological properties (G′, G″ and δ) observed among the samples group (Table 1). Figure 11 shows the elastic (G′), viscous (G″) and phase shift angle (δ) values for a constant strain rate frequency sweep test on a SA-PCL sample. The values of G′, G″ and δ of CG1-PCL was significantly lower compared to PEGDA-PCL and SA-PCL samples at 1 Hz frequency. Our rheological test data show that SA-PCL scaffold had the highest values of G′, G″ and δ compared to others.

## 4. Discussion

In this study, each hydrogel-PCL was stained for cell attachment, differentiation, proliferation, and stress fiber detection analysis. This was performed to determine whether the fabricated graft maintained certain conditions necessary for wound healing. For example, cell differentiation showed that fibroblasts were present in the tissues, and more specifically the presence of stress fibers and elongate shape of cells demonstrated that cells were under mechanical tension. Presence of DAPI-stained nuclei confirmed the presence of cells in each type of hydrogel. Cell proliferation in the hydrogels demonstrated that the environment was permissive to soluble signaling by the growth factors present in the serum. All these properties indicate that each scaffold could function as an in vivo dermal graft allowing host cells to populate the tissues and specialize into dermal fibroblasts with wound-healing capabilities.

This study found that PEDGA PCL nanofibers have good qualities regarding attachment of HDF cells. Cell culture assays found proliferation of cells and cell differentiation was also present. The amount of cells proliferating and differentiating was not as high as expected due to the lack of sufficient porosity on the scaffold. Physical characterization of the PEGDA PCL NFM dermal equivalent showed good absorption and degradation properties, therefore warranting further research to improve porosity in the PEGDA hydrogel. Our future goal is to micro-machine 3D PEGDA architecture and then coat PEGDA with PCL NFM to produce a PEGDA-PCL scaffold.

In this study, SA hydrogels were able to integrate with PCL NFM to improve the biological performance of the SA hydrogel. The successful integration between hydrogel and nanofiber matrix was enhanced by fibroblast cell culturing on the coupled polycaprolactone (PCL) and hydrogel constructs. Cell adhesion on PCL is important because it allows for cell spreading, migration, viability, and growth [37]. PCL is made using a precision deposition system that allows for the scaffold to have precise pore sizes and porosity, which promotes an increase in cell adhesion [38,39]. The authors’ previous studies found that that SA hydrogels without PCL fibers disintegrated and become more porous after two weeks of cell culture [40]. The increased porosity of SA with PCL after 2 weeks of cell culture led to a cell number increase for the SA hydrogel with PCL NFM. This explanation is in agreement with O’Brien et al. [41]. The authors found a strong correlation between the scaffold-specific surface area and cell attachment indices. The cell attachment and viability were primarily influenced by the scaffold-specific surface area of pore sizes for MC3T3 cells.

Based on the cell proliferation and differentiation assays, we conclude that CG1-PCL scaffold is the best choice among all the other test models used for this study. There are several reasons to use CG1 with PCL over others. First, the skin itself contains collagen I which is the main structural protein found in the extracellular matrix of the skin. Second, anchored collagen with and without the addition of PCL fibers permits fibroblasts to generate tension. This indicates that the dermal equivalent produced with collagen and PCL fibers could also restore senses such as touch to the affected wound area. Third, CG1-PCL has lower wettability and degradability compared to PEGDA-PCL and SA-PCL that makes it suitable for a dermal equivalent. Fourth, PCL nanofibers can provide adequate mechanical stability to CG1 for qualifying the resultant CG1-PCL as a skin graft. Finally, it is not possible to suture CG1 scaffold with skin. However, CG1-PCL graft can be sutured onto native skin during skin repair surgery. An in vivo evaluation of electrospun PCL graft for anterior cruciate ligament found that PCL graft can be sutured with native ACL and it can reconstruct the rat ACL [42]. Additionally, the study shows that PCL graft can facilitate induction of cells and collagen deposition in the graft.

When comparing the rheological properties of PEGDA-PCL, SA-PCL and CG1-PCL to similar properties of hyaluronic acid dermal fillers [43], most commonly used as the dermal equivalent model, the elastic modulus value (G’) of CG1-PCL (~120 Pa) is close to Restylane (294 Pa), Perlane (338 Pa), Juvéderm Ultra (111 Pa), Juvéderm Ultra Plus (159 Pa) and Juvéderm Voluma (220 Pa). Our rheological results revealed that the firmness of PEGDA-PCL (~5.34 Pa) and SA-PCL (~14.38 Pa) hydrogel were much higher than the dermal filler gels. 

According to the author’s knowledge, this is the first study to compare the effect of combining PCL with PEGDA, SA and CG1 on their physical and biological properties. The main purpose of applying PCL NFM on each of the hydrogels was that PCL membranes could serve as a reservoir for the supplies of nutrient in each of the scaffolds for fibroblast cell growth. It is possible to attach extracellular matrix proteins with PCL nanofibers that can enhance the cell growth in a PCL NFM incorporated scaffold [40,44]. The combined application of growth factor-immobilized nanofibers blended on a hydrogel can further enhance the biocompatibility of hydrogel, which is a potential area of new research. Another future research is to evaluate the reinforcement of hydrogel by nanofiber mesh with a goal to suture the hydrogel to native skin for skin repair. 

The values of the biological properties of the PCL fiber-coated PEGDA in this study were different from our previous studies [36], likely because we used a different architecture of PCL NFM and a different cell line in this study. Kotturi et al. [36] used 12 layers of PCL NFM to produce PEGDA-PCL samples, whereas this study used 24 layers of PCL NFM. This study used a higher number of fiber layers because our previous studies observed a correlated higher amount of cellular attachment and proliferation for PCL membrane with a higher number of fiber layers [45]. Kotturi et al. [36] studied how human hepatocellular carcinoma cells (GS5 cells) adhered, proliferated, and migrated higher in the PEGDA-PCL scaffold than in PEGDA alone. This study (using human fibroblasts) also found that fibroblasts adhered, proliferated, and differentiated in the PEGDA-PCL scaffold. The results of the PCL fiber-coated SA in this study supplements the authors’ previous studies [40], where the authors examined osteoblast (MT3T3E1) cell viability in a sodium alginate (SA) hydrogel and measured the adhesion strength between polycaprolactone (PCL) and SA hydrogel scaffolds after two weeks of culture. A commercial PCL scaffold (3D Insert™-PCL scaffolds, 3D Biotek Inc., Hillsborough, NJ, USA) was used in the previous study, whereas this study used electrospun PCL nanofibers. Although the cell lines were different, both studies found excellent biocompatibility on the scaffolds and cell migration from SA hydrogel to PCL layers. The values of the biological properties of the PCL fiber-coated CG1 in this study were different from other author studies [44,46], likely because this study used a unique PCL NFM architecture and CG1 composition for making CG1-PCL scaffold. Furthermore, the CG1-PCL fabrication process was different from other studies. In this study, the PCL-NFM was attached on a plastic ring and then cell-seeded CG1 solution was cured on the hole of the plastic ring along with the PCL NFM. 

The study was limited to investigating influence of coating PEGDA, SA, and CG-1 with PCL on the key physical, mechanical, and biological properties of composite scaffold for skin tissue engineering application. This study was unable to compare the effect of addition of PCL on PEGDA, SA and CG1 on the thermal properties. Our research group have recently conducted thermal analysis on PCL during the study of using PCL nanofiber mesh in a face mask application [47]. We have found that PCL is stable in extreme conditions and no plasticizing effect occurs due to the presence of a solvent. In future, the viscoelastic properties at different temperature of both SA and CG1 due to addition of PCL NFM will be analyzed to evaluate integrity of the scaffold at 25 and 37 °C. We have recently conducted in vivo biocompatibility study of CG1-PCL scaffold using a rat model. Our study results concluded that CG1-PCL is biocompatible. The results will be published in another literature in near future. Human fibroblast-seeded collagen scaffolds without and with PCL fibers showed existence of stress fibers and elongate cell structure, suggesting tension in the gel. However, the difference of tension generation values between fibroblast-seeded CG1 and CG1-PCL scaffolds is still unknown and warrants further investigation.

## 5. Conclusions

The objectives included determining the in vitro biological efficacy of PEGDA-PCL, SA-PCL, and CG1-PCL using human dermal fibroblasts. The study conducted cell adhesion, proliferation, and differentiation using standard cell culture and staining assays. In addition, morphological and degradation properties of each scaffold were compared. This study found the following:

1. PEDGA-PCL scaffold had good qualities regarding attachment of HDF cell to nanofibers. Physical characterization of PEGDA-PCL showed good absorption and degradation properties.
2. SA-PCL had measurable physical and biological characteristics. Cell viability was exceptional where HDF cells satisfactorily differentiated inside the SA hydrogel.
3. CG1-PCL had better biological and mechanical characteristics as a skin graft compared to PEGDA-PCL and SA-PCL.

The translation of this study will be in vivo studies of CG1-PCL graft to verify whether CG1-PCL graft is suitable for skin repair.

## Figures and Tables

**Table 1:** Difference between viscoelastic properties between PEGDA-PCL, SA-PCL and CG1-PCL (*n* = 3).

Experimental Parameters	PEGDA-PCL	SA-PCL	CG1-PCL
Viscous modulus G″ (kPa)	0.53 ± 0.18	6.58 ± 3.41	0.002 ± 0.001
Elastic modulus, G′ (kPa)	5.34 ± 0.23	14.82± 1.92	0.12 ± 0.01
Phase shift angle, δ (degree)	5.68 ± 0.22	16.85± 6.29	1.14 ± 0.11

## Data Availability

Essential data are contained within the article. The raw data are available on request from the corresponding author.

## References

[1] Guo S., Dipietro L.A. (2010). Factors affecting wound healing. J. Dent. Res..

[2] Sen C.K. (2019). Human Wounds and Its Burden: An Updated Compendium of Estimates. Adv. Wound Care.

[3] Wang D.-A., Williams C., Yang F., Elisseeff J. (2004). Enhancing the tissue-biomaterial interface: Tissue-initiated integration of biomaterials. Adv. Funct. Mater..

[4] Khandaker M., Orock A., Tarantini S., White J., Yasar O. (2016). Biomechanical Performances of Networked Polyethylene Glycol Diacrylate: Effect of Photoinitiator Concentration, Temperature, and Incubation Time. Int. J. Biomater..

[5] Progri H., Tummala S., Kotturi H., Williams W., Khandaker M. In vivo evaluation of novel PEGDA-PCL scaffold for cartilage generation. Proceedings of the 2019 Orthopadic Research Society Technical Meeting.

[6] Cole M.A., Quan T., Voorhees J.J., Fisher G.J. (2018). Extracellular matrix regulation of fibroblast function: Redefining our perspective on skin aging. J. Cell Commun. Signal.

[7] Lee K.Y., Mooney D.J. (2012). Alginate: Properties and biomedical applications. Prog. Polym. Sci..

[8] Chen F., Tian M., Zhang D.M., Wang J.Y., Wang Q.G., Yu X.X., Zhang X.H., Wan C.X. (2012). Preparation and characterization of oxidized alginate covalently cross-linked galactosylated chitosan scaffold for liver tissue engineering. Mater. Sci. Eng. C.

[9] Gleghorn J.P., Lee C.S., Cabodi M., Stroock A.D. (2008). Adhesive properties of laminated alginate gels for tissue engineering of layered structures. J. Biomed. Mater. Res.—Part A.

[10] Melrose J., Smith S., Ghosh P., Taylor T.K. (2001). Differential Expression of Proteoglycan Epitopes and Growth Characteristics of Intervertebral Disc Cells Grown in Alginate Bead Culture. Cells Tissues Organs.

[11] Lee C.S., Gleghorn J.P., Won Choi N., Cabodi M., Stroock A.D. (2007). Integration of layered chondrocyte-seeded alginate hydrogel scaffolds. Biomaterials.

[12] Drury J.L., Dennis R.G., Mooney D.J. (2004). The tensile properties of alginate hydrogels. Biomaterials.

[13] Ramaswamy S., Wang D.A., Fishbein K.W., Elisseeff J.H., Spencer R.G. (2006). An analysis of the integration between articular cartilage and nondegradable hydrogel using magnetic resonance imaging. J. Biomed. Mater. Res. Part B-Appl. Biomater..

[14] Lodish H., Berk A., Zipursky S.L., Matsudaira P., Baltimore D., Darnell J., Freeman W.H. (2000). Collagen: The Fibrous Proteins of the Matrix. Molecular Cell Biology.

[15] Albu M.G., Titorencu I., Ghica M.V., Pignatello R. (2011). Collagen-Based Drug Delivery Systems for Tissue Engineering, Biomaterials. Applications for Nanomedicine.

[16] Kanungo I., Fathima N.N., Rao J.R., Nair B.U. (2013). Influence of PCL on the material properties of collagen based biocomposites and in vitro evaluation of drug release. Mater. Sci. Eng. C Mater. Biol. Appl..

[17] Scaffaro R., Maio A., Lopresti F., Botta L. (2017). Nanocarbons in Electrospun Polymeric Nanomats for Tissue Engineering: A Review. Polymers.

[18] Scaffaro R., Lopresti F. (2018). Properties-morphology relationships in electrospun mats based on polylactic acid and graphene nanoplatelets. Compos. Part A Appl. Sci. Manuf..

[19] Scaffaro R., Lopresti F., Botta L. (2017). Preparation, characterization and hydrolytic degradation of PLA/PCL co-mingled nanofibrous mats prepared via dual-jet electrospinning. Eur. Polym. J..

[20] Scaffaro R., Lopresti F., Maio A., Botta L., Rigogliuso S., Ghersi G. (2017). Electrospun PCL/GO-g-PEG structures: Processing-morphology-properties relationships. Compos. Part A Appl. Sci. Manuf..

[21] Scaffaro R., Lopresti F., D’Arrigo M., Marino A., Nostro A. (2018). Efficacy of poly(lactic acid)/carvacrol electrospun membranes against Staphylococcus aureus and Candida albicans in single and mixed cultures. Appl. Microbiol. Biotechnol..

[22] Khandaker M., Riahinezhad S., Jamadagni H.G., Morris T.L., Coles A.V., Vaughan M.B. (2017). Use of Polycaprolactone Electrospun Nanofibers as a Coating for Poly (methyl methacrylate) Bone Cement. Nanomaterials.

[23] Khandaker M., Riahinezhad S. (2016). Process and Apparatus to Create 3D Tissue Scaffold Using Electrospun Nanofiber Matrix and Photosensitive Hydrogel, USA. https://patents.google.com/patent/WO2017147183A1/en.

[24] Nazarnezhad S., Baino F., Kim H.W., Webster T.J., Kargozar S. (2020). Electrospun Nanofibers for Improved Angiogenesis: Promises for Tissue Engineering Applications. Nanomaterials.

[25] Bazzolo B., Sieni E., Zamuner A., Roso M., Russo T., Gloria A., Dettin M., Conconi M.T. (2021). Breast Cancer Cell Cultures on Electrospun Poly(ε-Caprolactone) as a Potential Tool for Preclinical Studies on Anticancer Treatments. Bioengineering.

[26] Abebayehu D., Spence A.J., McClure M.J., Haque T.T., Rivera K.O., Ryan J.J. (2019). Polymer scaffold architecture is a key determinant in mast cell inflammatory and angiogenic responses. J. Biomed. Mater. Res. Part A.

[27] Dettin M., Zamuner A., Roso M., Gloria A., Iucci G., Messina G.M., D’Amora U., Marletta G., Modesti M., Castagliuolo I. (2015). Electrospun Scaffolds for Osteoblast Cells: Peptide-Induced Concentration-Dependent Improvements of Polycaprolactone. PLoS ONE.

[28] Bosworth L.A., Turner L.-A., Cartmell S.H. (2013). State of the art composites comprising electrospun fibres coupled with hydrogels: A review. Nanomed. Nanotechnol. Biol. Med..

[29] Zhang Y., Liu J., Huang L., Wang Z., Wang L. (2015). Design and performance of a sericin-alginate interpenetrating network hydrogel for cell and drug delivery. Sci. Rep..

[30] Vaughan M.B., Ramirez R.D., Andrews C.M., Wright W.E., Shay J.W. (2009). H-Ras Expression in Immortalized Keratinocytes Produces an Invasive Epithelium in Cultured Skin Equivalents. PLoS ONE.

[31] Vaughan M.B., Ramirez R.D., Brown S.A., Yang J.C., Wright W.E., Shay J.W. (2004). A reproducible laser-wounded skin equivalent model to study the effects of aging in vitro. Rejuvenation Res..

[32] Vaughan M.B., Odejimi T.D., Morris T.L., Sawalha D., Spencer C.L. (2014). A new bioassay identifies proliferation ratios of fibroblasts and myofibroblasts. Cell Biol. Int..

[33] Khandaker M., Riahinezhad S., Sultana F., Vaughan M.B., Knight J., Morris T.L. (2016). Peen treatment on a titanium implant: Effect of roughness, osteoblast cell functions, and bonding with bone cement. Int. J. Nanomed..

[34] Dugina V., Fontao L., Chaponnier C., Vasiliev J., Gabbiani G. (2001). Focal adhesion features during myofibroblastic differentiation are controlled by intracellular and extracellular factors. J. Cell Sci..

[35] Vaughan M.B., Howard E.W., Tomasek J.J. (2000). Transforming Growth Factor-β1 Promotes the Morphological and Functional Differentiation of the Myofibroblast. Exp. Cell Res..

[36] Kotturi H., Abuabed A., Zafar H., Sawyer E., Pallipparambil B., Jamadagni H., Khandaker M. (2017). Evaluation of Polyethylene Glycol Diacrylate-Polycaprolactone Scaffolds for Tissue Engineering Applications. J. Funct. Biomater..

[37] Izquierdo R., Garcia-Giralt N., Rodriguez M.T., Cáceres E., García S.J., Gómez Ribelles J.L., Monleón M., Monllau J.C., Suay J. (2008). Biodegradable PCL scaffolds with an interconnected spherical pore network for tissue engineering. J. Biomed. Mater. Res. Part A.

[38] Kim J.-Y., Park E.-K., Kim S.-Y., Shin J.-W., Cho D.-W. (2008). Fabrication of a SFF-based three-dimensional scaffold using a precision deposition system in tissue engineering. J. Micromech. Microeng..

[39] Zein I., Hutmacher D.W., Tan K.C., Teoh S.H. (2002). Fused deposition modeling of novel scaffold architectures for tissue engineering applications. Biomaterials.

[40] Khandaker M., Vaughan M., Starly B. (2013). The Influence of MgO Nanoparticles on the Osseointegration of Polycaprolactone-Sodium Alginate Hydrogel Interfaces. Curr. J. Appl. Sci. Technol..

[41] O’Brien F.J., Harley B.A., Yannas I.V., Gibson L.J. (2005). The effect of pore size on cell adhesion in collagen-GAG scaffolds. Biomaterials.

[42] Petrigliano F.A., Arom G.A., Nazemi A.N., Yeranosian M.G., Wu B.M., McAllister D. (2015). In vivo evaluation of electrospun polycaprolactone graft for anterior cruciate ligament engineering. Tissue Eng. Part A.

[43] Stocks D., Sundaram H., Michaels J., Durrani M.J., Wortzman M.S., Nelson D.B. (2011). Rheological evaluation of the physical properties of hyaluronic acid dermal fillers. J. Drugs Dermatol..

[44] Gentile P., McColgan-Bannon K., Gianone N.C., Sefat F., Dalgarno K., Ferreira A.M. (2017). Biosynthetic PCL-graft-Collagen Bulk Material for Tissue Engineering Applications. Materials.

[45] Sultana F., Vaughan M., Khandaker M., Korach C., Tekalur S., Zavattieri P. (2017). Effect of Fiber Architecture on the Cell Functions of Electrospun Fiber Membranes. Mechanics of Biological Systems and Materials, Volume 6: Proceedings of the 2016 Annual Conference on Experimental and Applied Mechanics.

[46] Augustine R., Kalarikkal N., Thomas S. (2014). Advancement of wound care from grafts to bioengineered smart skin substitutes. Prog. Biomater..

[47] Khandaker M., Progri H., Arasu D.T., Nikfarjam S., Shamim N. (2021). Use of Polycaprolactone Electrospun Nanofiber Mesh in a Face Mask. Materials.

